# A protocol for organoids from the gynecomastia patients

**DOI:** 10.3389/fbioe.2025.1593368

**Published:** 2025-08-13

**Authors:** Fangjian Shang, Zihao Li, Ji Feng, Qi Wang, Mengyang An, Zengren Zhao, Bo Liu

**Affiliations:** The First Hospital of Hebei Medical University, Shijiazhuang, Hebei, China

**Keywords:** gynecomastia, organoid culture, immunohistochemistry and immunofluorescence analysis, qPCR validation, 3D disease modeling, hormone receptor signaling

## Abstract

**Background:**

Gynecomastia, characterized by benign proliferation of male breast glandular tissue, is a prevalent condition with complex etiologies. However, the absence of effective *in vitro* models has hindered mechanistic investigations and therapeutic development.

**Methods:**

In this study, we established and characterized organoids derived from the breast tissues of six male gynecomastia patients, including physiological, idiopathic, and hormone-related subtypes. Organoid fidelity was evaluated using hematoxylin and eosin (H&E) staining, immunohistochemistry (IHC), immunofluorescence (IF), and quantitative PCR (qPCR), targeting a panel of lineage-specific and proliferative markers.

**Results:**

The organoids recapitulated key histological and molecular features of their corresponding source tissues, including epithelial architecture and expression of CK14, CK18, Ki67, and ERα. Marker expression was generally consistent between organoids and tissues at both the protein and transcriptional levels. Notably, ERα protein levels were reduced in organoids, while ESR1 mRNA expression remained stable, suggesting post-transcriptional regulation related to culture conditions.

**Conclusion:**

Our study presents a practical and reproducible protocol for generating gynecomastia-derived organoids and highlights their utility as a disease-relevant platform for future research in male breast pathology and hormone-related mechanisms.

## 1 Introduction

Gynecomastia, or benign proliferation of male breast glandular tissue, represents one of the most prevalent breast disorders among men, with reported incidence rates ranging from 30% to 65% in the general male population. The condition may occur across all age groups and frequently presents as bilateral, concentric breast enlargement centered around the nipple-areolar complex.

This condition can affect males of all ages, primarily presenting as bilateral involvement, although unilateral cases may also occur. Clinically, it manifests as breast enlargement, typically distributed concentrically and symmetrically from the nipple outward. The causes of male gynecomastia are multifactorial and can generally be categorized as hormone-related, idiopathic, or physiological (Cuhaci et al., 2014).

Beyond physical discomfort, gynecomastia imposes considerable psychosocial burdens on affected individuals, including diminished self-esteem, social anxiety, and professional embarrassment. The visible feminization of the male chest often results in psychological distress and impaired quality of life (Rew et al., 2015). Understanding the underlying pathophysiological mechanisms of gynecomastia is essential for the development of targeted treatments and improved patient care. However, progress in this field has been hampered by the lack of physiologically relevant *in vitro* models that faithfully replicate human male breast tissue architecture and hormone responsiveness.

Recent advances in organoid technology have enabled the development of three-dimensional (3D) culture systems that closely mimic native tissue architecture and function. These self-organizing structures retain essential features of their tissue of origin, including cellular heterogeneity, spatial organization, and molecular signaling, making them ideal for disease modeling and therapeutic screening (Zhao et al., 2022).

In this study, we aimed to establish a standardized protocol for generating and maintaining patient-derived organoids from male gynecomastia tissues. We further sought to characterize their structural and molecular fidelity to source tissues, defined as the preservation of key epithelial (CK18, CK14), proliferative (Ki67), and hormonal (ERα, PR, HER2) markers. These were primarily assessed via immunohistochemistry (IHC), with additional validation of selected markers (CK18, CK14, Ki67, ERα) by immunofluorescence (IF), and transcript-level confirmation of KRT14, MKI67, and ESR1 via qPCR.

Unlike previous studies focused on female breast tissue, this study is the first to establish organoids derived from male gynecomastia, providing a sex-specific model for hormone-driven benign breast pathology.

## 2 Methods

### 2.1 Patient samples and ethical approval

**FIGURE 1:**
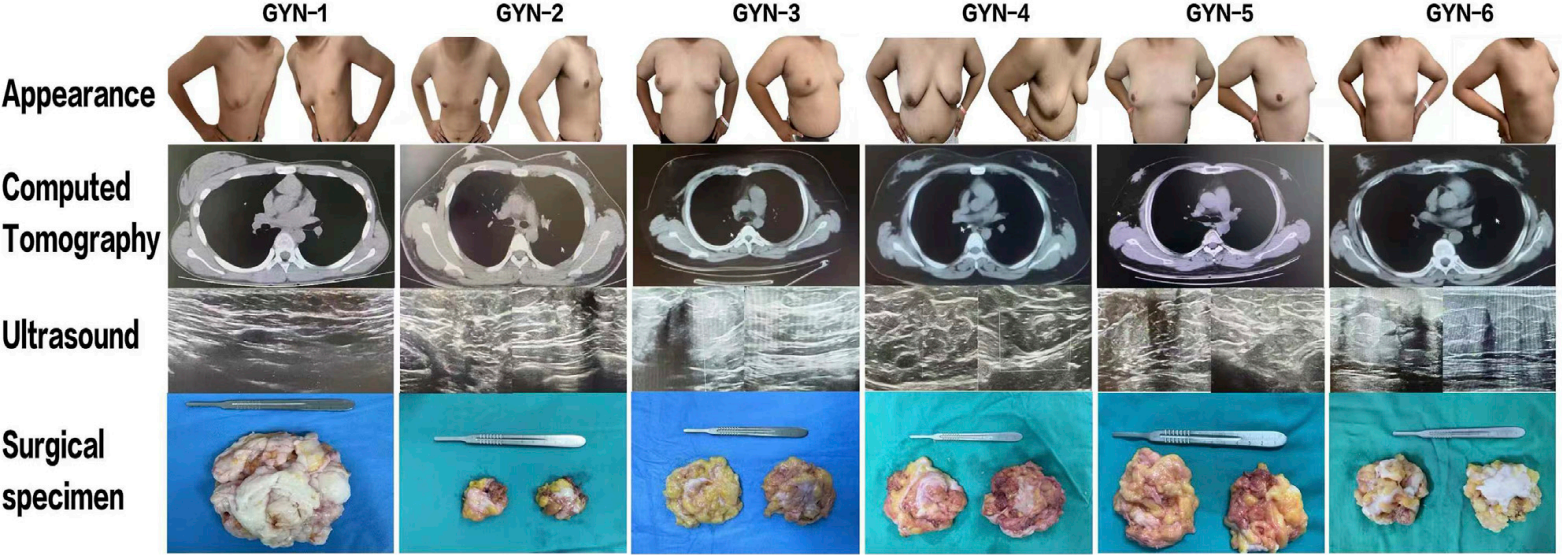
Preoperative evaluation and gross specimen collection from six gynecomastia patients. Preoperative frontal and lateral photographs, CT and ultrasound images, and intraoperative glandular specimens are shown. Note: Frontal image for GYN-1 was not available due to clinical documentation limitations; only lateral images were retained.

**FIGURE 2:**
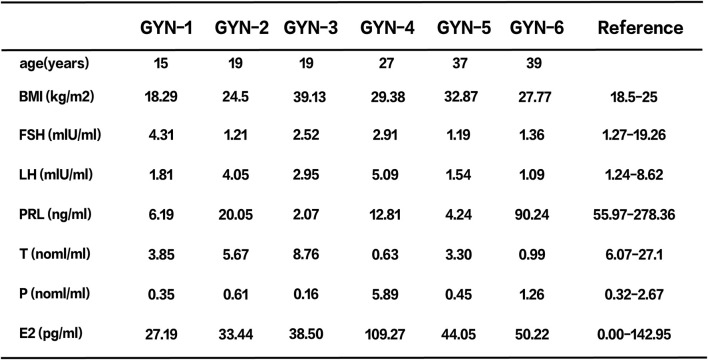
Basic characteristics and hormone levels of six patients.

Breast gland tissues were obtained from six male patients diagnosed with gynecomastia between March and July 2024 at the First Hospital of Hebei Medical University. The cohort included one physiological case (unilateral), two idiopathic cases, and three hormone-related cases (Figure 1). Clinical data including age, body mass index (BMI), and serum hormone levels—follicle-stimulating hormone (FSH), luteinizing hormone (LH), prolactin (PRL), testosterone (T), progesterone (P4), and estradiol (E2)—were recorded (Figure 2).

### 2.2 Inclusion criteria

1. Confirmed clinical diagnosis of gynecomastia by physical examination, ultrasonography, and MRI;
2. Underwent endoscopic subcutaneous mastectomy with pathological confirmation of diagnosis;
3. Complete preoperative clinical and laboratory data available;
4. Absence of infectious diseases or history of recent hormonal medication use.

All surgical procedures were performed using the minimally invasive Liu and Shang two-port, seven-step technique, optimized for cosmetic outcomes (Shang et al., 2023). Ethical approval was obtained from the institutional review board (Approval No. S00980), and informed consent was secured from all participants.

### 2.3 Pre-experimental preparation

All procedures were conducted in a biosafety level 2 (BSL-2) laminar flow cabinet to maintain aseptic conditions.

• Culture Environment: 37°C humidified incubator with 5% CO_2_.
• Reagents and Materials: Detailed in Supplementary Table S1. The organoid culture medium was based on previously published formulations (Sachs et al., 2018)with minor modifications, and included EGF, FGF7, FGF10, and other supplements optimized for male breast tissue.

### 2.4 Primary culture of male gynecomastia organoids

#### 2.4.1 Sample collection and handling

**FIGURE 3:**
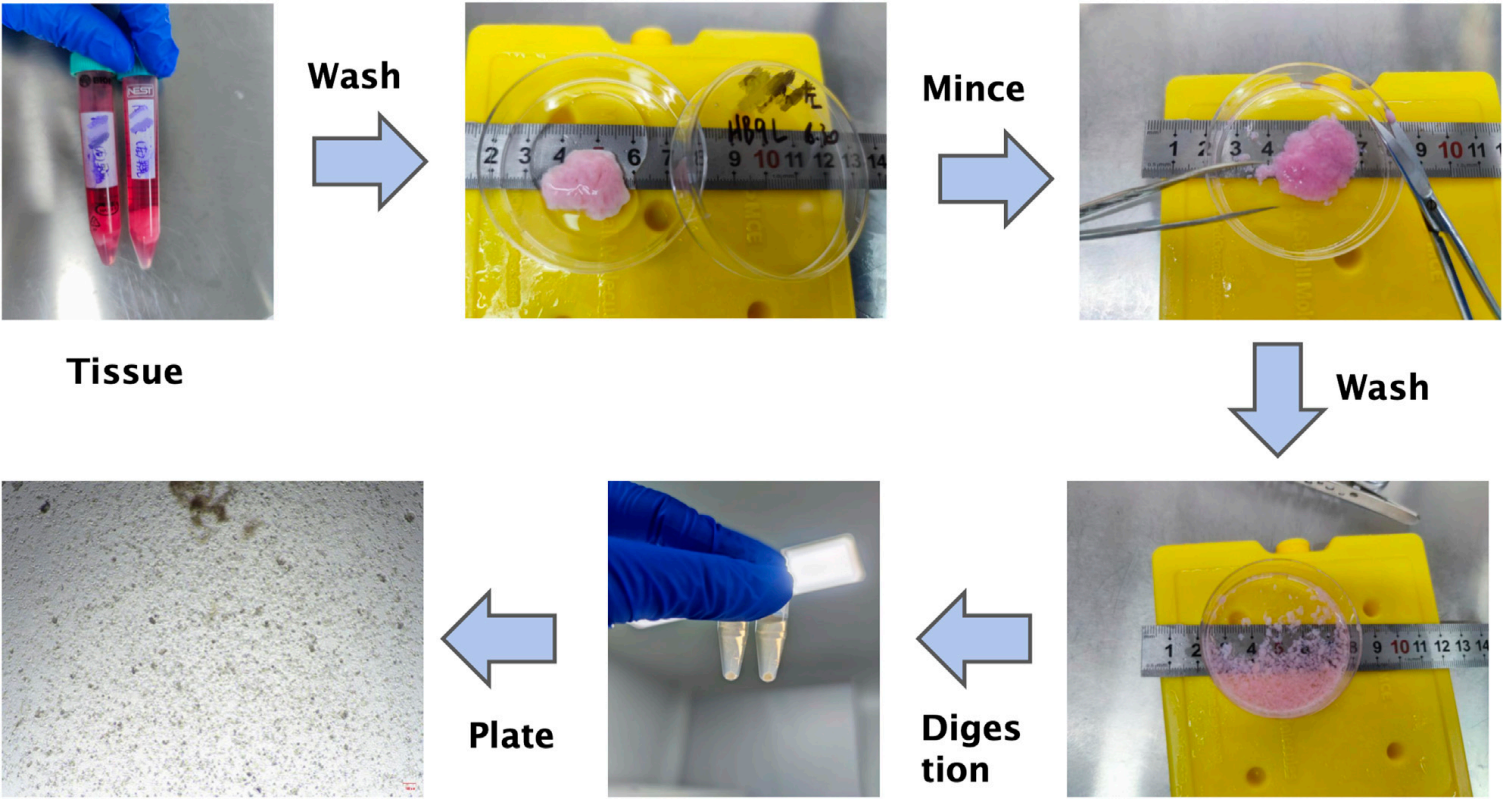
Schematic workflow of primary organoid culture from male gynecomastia tissues. This step-by-step diagram summarizes tissue processing, enzymatic digestion, filtration, Matrigel embedding, and culture maintenance. Note: This schematic complements the detailed protocol provided in the Methods section.

The overall workflow for organoid derivation from gynecomastia tissues is illustrated in Figure 3. Freshly excised gynecomastia tissues were immediately placed in tissue preservation solution (OM45) and transported on ice to the laboratory as quickly as possible to maintain tissue viability and minimize exposure to ambient temperatures.

The OM45, OM41, OM43, and OM21 solutions were prepared according to the protocol published by Sachs et al. (Sachs et al., 2018), which established a living biobank of breast organoids. Specifically, OM45 was used as tissue preservation buffer, OM41 as enzymatic digestion solution, OM43 as neutralizing buffer, and OM21 referred to cold Matrigel used for embedding.

#### 2.4.2 Pretreatment and washing

The tissue was transferred to a sterile petri dish, and necrotic or adipose tissue was carefully removed using sterile surgical instruments to retain as much glandular tissue as possible.

Upon transfer to a sterile dish, necrotic tissue (appearing dark, friable, or poorly cohesive) and yellowish adipose tissue were gently excised using ophthalmic scissors and forceps. The remaining pale, dense, fibrous glandular tissue was retained and processed for culture. The identification was based on visual assessment of firmness and gross appearance under sterile conditions.

Next, the processed tissue was placed in a 50 mL centrifuge tube with 10–15 mL of PBS, gently mixed, and centrifuged. This washing step was repeated until the supernatant appeared clear, ensuring the removal of blood and debris to create a clean culture environment.

#### 2.4.3 Tissue mincing and digestion

• Mincing: The washed tissue was transferred to a petri dish and minced into ∼1 mm^3^ fragments using ophthalmic scissors. These fragments were then placed in a 15 mL centrifuge tube with 10 mL of cold PBS, centrifuged at 1,200 rpm at 25°C for 3 min, and the supernatant was discarded.
• Digestion: 1 mL of tissue digestion solution (OM41) was added to the tube. After gentle mixing, the contents were transferred to a 1.5 mL centrifuge tube and incubated in a 37°C shaker for 30 min. Microscopic evaluation was performed every 10 min to monitor the presence of single cells or small clusters, and digestion time was adjusted based on tissue density and enzyme activity to avoid over-digestion.
• Centrifugation and Neutralization: After digestion, the cells were centrifuged, the supernatant discarded, and 1 mL of neutralizing solution (OM43) was added and gently mixed.

### 2.5 Critical: digestion time should be adjusted according to tissue texture and enzyme efficiency

#### 2.5.1 Cell extraction and purification

• After standing for 3 min, the supernatant was removed and the pellet retained.
• PBS was added and gently mixed. The suspension was passed through a 100 μm cell strainer to remove large debris, and the filtrate was collected into a 15 mL tube.
• The sample was then centrifuged at 1,300 rpm for 5 min at 4°C to collect the cell pellet.

#### 2.5.2 (Optional) red blood cell lysis

If red blood cells were abundant, the pellet was incubated in red blood cell lysis buffer for 2 min, followed by a PBS wash to ensure the organoid culture was free of contaminants.

#### 2.5.3 Organoid seeding

• The cell pellet was mixed with an equal volume of cold Matrigel (OM21).
• Using a 200 μL pipette, 70 μL droplets of the Matrigel-cell mixture were dispensed into each well of a 24-well plate.
• The plate was incubated at 37°C with 5% CO_2_ for 30 min to allow the Matrigel to solidify, after which 500 μL of prewarmed organoid culture medium was added to each well.

#### 2.5.4 Organoid culture

• The plates were maintained in a humidified incubator at 37°C with 5% CO_2_, and the culture medium was replaced every 2–3 days to avoid disturbing the Matrigel structure.
• Organoid formation was observed within 4–6 days. Structures with diameters greater than 100 μm were considered successfully cultured organoids.

#### 2.5.5 Organoid observation

**FIGURE 4:**
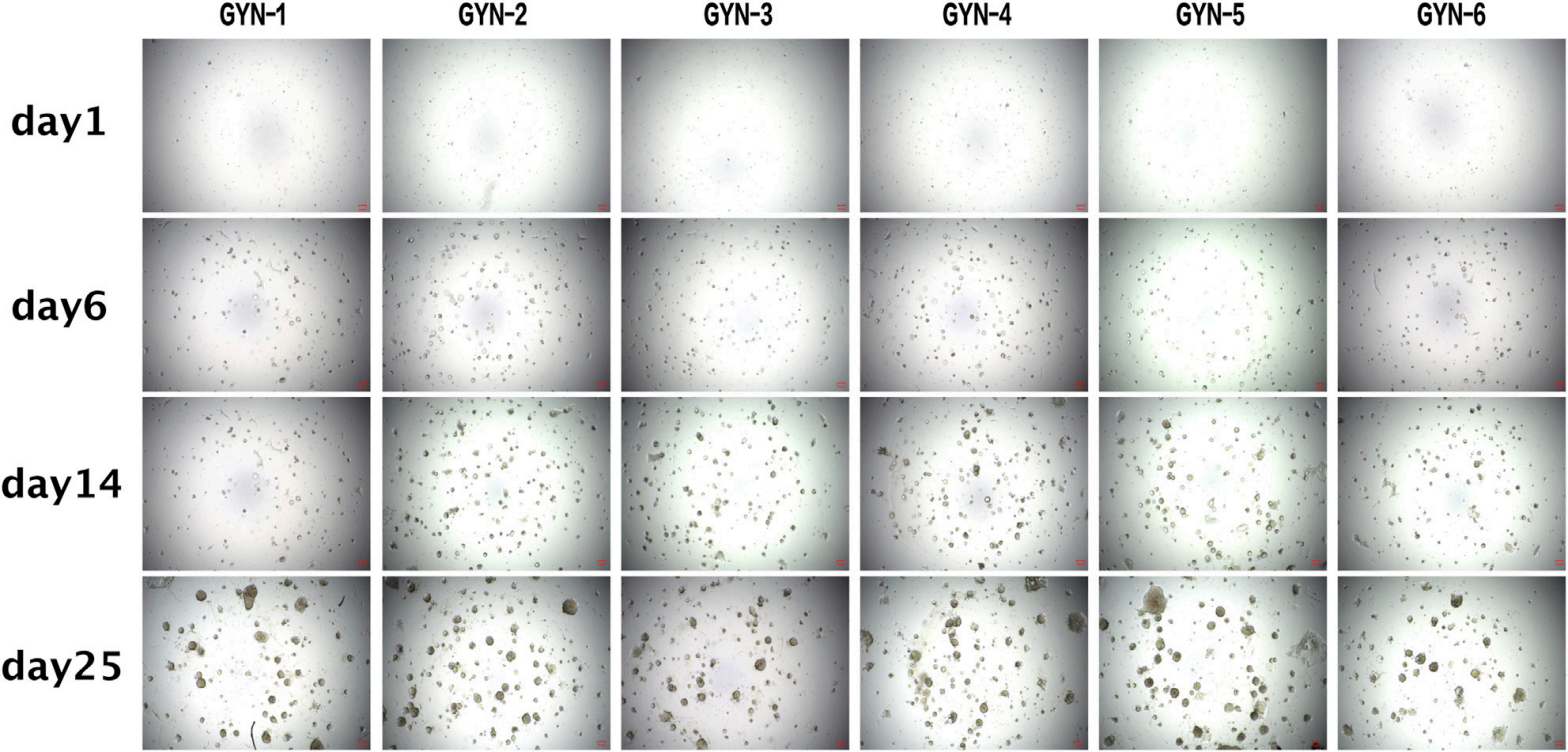
Morphological observation of gynecomastia organoids over extended culture periods. Brightfield images of organoids derived from six patient samples at Days 1, 6, 14, and 25, demonstrating gradual development of spheroid and gland-like structures. Scale bar = 100 μm. All image panels now include scale bars to enable consistent visual comparison across samples. Quantitative measurements of organoid size were not included in this figure due to variability in organoid shape and limitations in standardizing diameter measurements from 2D images.

**FIGURE 5:**
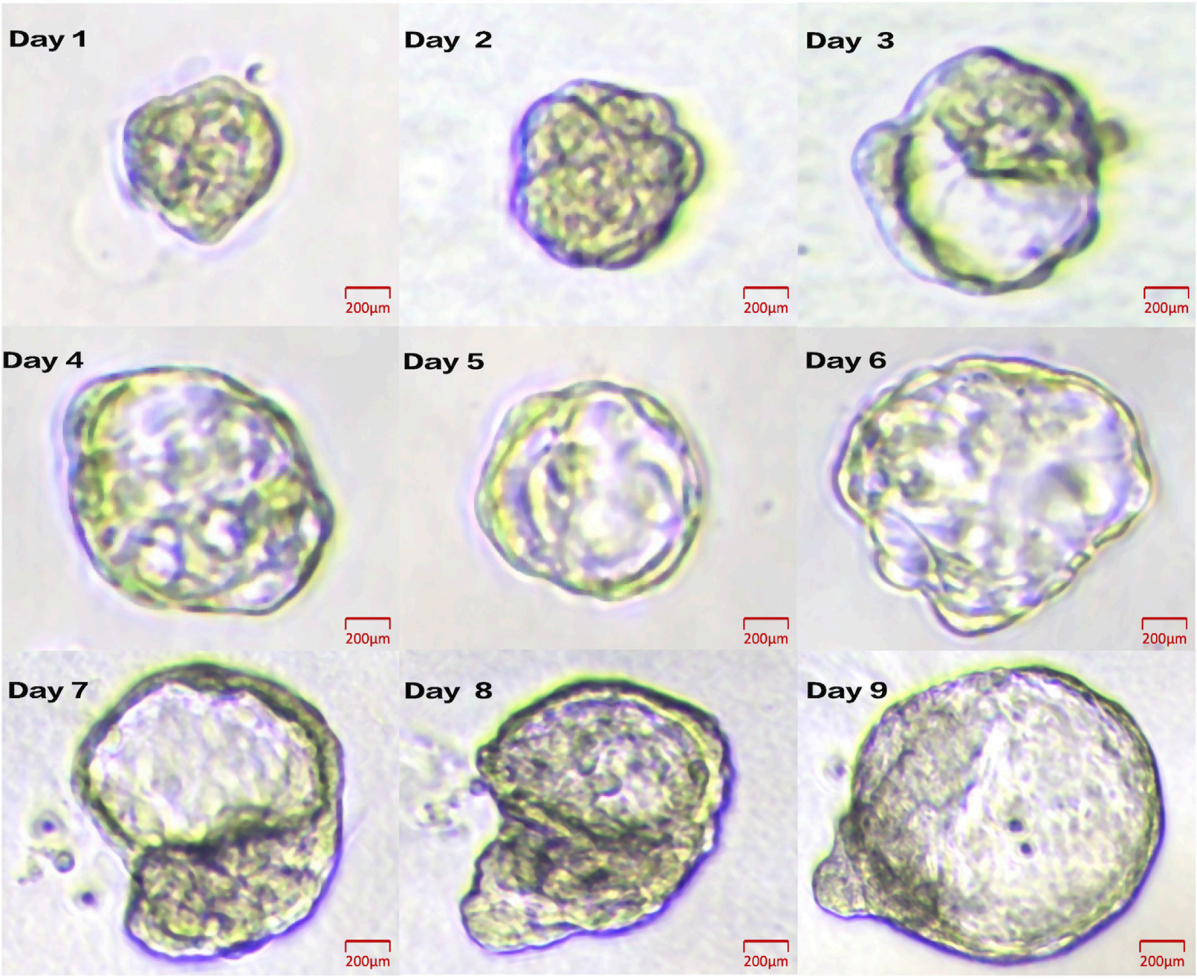
Daily growth dynamics of a representative organoid from GYN-1. Brightfield images show progressive morphological changes from Day 1 to Day 9. Scale bar = 100 μm.

• Representative images of organoid droplets were captured to assess morphology, proliferation status, and the absence of contamination (Figure 4).
• One representative organoid derived from GYN-1 was selected for daily microscopic observation over a 9-day period (Figure 5), documenting its structural changes, including boundary expansion and microstructure development. This supports the stability and reproducibility of the culture system and establishes a foundation for further functional analyses.

### 2.6 Passaging of gynecomastia organoids

#### 2.6.1 Organoid collection

**FIGURE 6:**
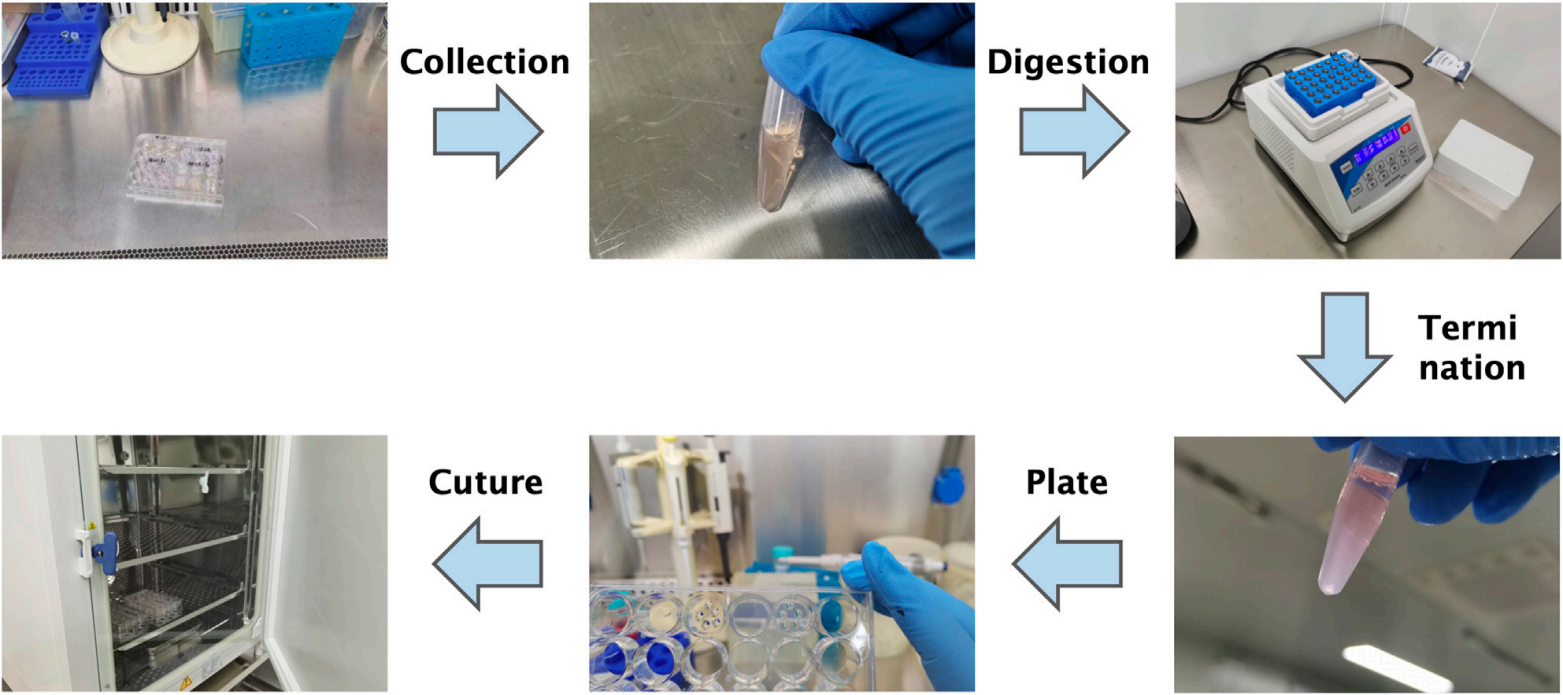
Schematic overview of the organoid passaging process. Diagram summarizes mechanical disruption, enzymatic digestion, re-embedding in Matrigel, and reseeding. Note: Refer to the Methods section for detailed protocols.

The passaging procedure for organoids is shown in Figure 6. The Matrigel surrounding the organoids was gently disrupted using a pipette. The organoid-containing suspension was transferred to a 1.5 mL centrifuge tube and briefly centrifuged using a handheld centrifuge for 30 s. The supernatant was discarded, and the pellet was retained).

#### 2.6.2 Organoid digestion

500 μL of gentle cell dissociation reagent (OM43) was added to the tube, sealed with parafilm, and incubated on a 37°C orbital shaker for 10 min. Digestion progress was monitored under an inverted microscope, and the duration was adjusted according to organoid structure. The reaction was stopped once single cells or small clusters were observed.

#### 2.6.3 Stopping digestion

The sample was centrifuged using a handheld centrifuge, the supernatant discarded, and the pellet resuspended in 1 mL of DMEM. Gentle pipetting ensured a uniform cell suspension.

## 3 Cell seeding into culture plates

Following another low-speed centrifugation, the pellet was mixed with an equal volume of Matrigel. After thorough mixing, five 10 μL droplets were dispensed into each well of a 24-well plate. After polymerization at 37°C for 30 min, 500 μL of organoid culture medium was added to each well.

### 3.1 Histology and immunohistochemistry

Organoids and matched source tissues were fixed in 4% paraformaldehyde for 24 h, processed via standard paraffin embedding, and sectioned at 4 μm thickness. H&E staining followed conventional protocols for evaluating structural morphology.

For IHC, antigen retrieval was performed using citrate buffer (pH 6.0) under high pressure. Endogenous peroxidase was blocked using 3% H_2_O_2_, and non-specific binding was blocked with 5% bovine serum albumin (BSA). Primary antibodies (CK14, CK18, Ki67, ERα, etc.) were incubated overnight at 4°C. Visualization was performed using DAB, and nuclei were counterstained with hematoxylin.

### 3.2 Immunofluorescence staining

Sections were permeabilized with 0.2% Triton X-100, blocked with 5% BSA, and incubated with primary antibodies targeting CK18, CK14, Ki67, and ERα overnight at 4°C. Alexa Fluor-conjugated secondary antibodies were used for detection, and nuclei were counterstained with DAPI. Images were acquired using a fluorescence microscope. Quantification of fluorescence intensity was performed using Fiji (ImageJ). Among the six cultured organoids, GYN-1 (physiological subtype) and GYN-4 (hormone-related subtype) were selected for immunofluorescence analysis based on their distinct clinical classification and the quality and reproducibility of their staining results.

### 3.3 Statistical analysis

• IHC Quantification: For each patient-derived organoid and matched tissue sample, three non-overlapping regions of interest (ROIs) were selected under 200× magnification. Each ROI covered an area of approximately 0.05 mm^2^, typically encompassing 80–120 cells. The DAB signal intensity for each ROI was quantified using ImageJ (Color Deconvolution → H-DAB mode), and the grayscale values were averaged to obtain a patient-level mean for each marker.

To allow relative comparison across different markers and groups, intensity values were normalized by dividing each sample’s mean by the overall average intensity of that marker across all samples, yielding normalized relative expression values for graphical representation.

• IF Quantification: IF Quantification: For each sample (organoid and matched tissue), three non-overlapping fields (at 200× magnification) were randomly selected for quantification. Each field covered ∼0.05 mm^2^ and included approximately 80–120 cells. Fluorescence intensities were measured for CK18, CK14, Ki67, and ERα using Fiji (ImageJ) with consistent exposure settings across all images. Intensities from the three fields were averaged to obtain per-sample mean values. These were further normalized relative to the overall mean for each marker across samples to allow inter-group comparison. Individual field-level values are provided in Supplementary Table S2.
• Statistical comparisons between organoids and paired tissues were conducted using paired t-tests in GraphPad Prism 9.5. Subgroup comparisons were performed across physiological (GYN-1), idiopathic (GYN-2, GYN-3), and hormone-related (GYN-4, GYN-5, GYN-6) cases.

### 3.4 Quantitative real-time PCR analysis

To validate the transcriptional fidelity between organoids and their source tissues, quantitative real-time PCR (qPCR) was performed on samples from two representative cases: GYN-1 (physiological) and GYN-4 (hormone-related). Total RNA was extracted from organoid and matched tissue samples using TRIzol reagent (Invitrogen, United States) according to the manufacturer’s protocol. Reverse transcription was carried out with the PrimeScript™ RT reagent kit (Takara, Japan), and qPCR was conducted using SYBR Green Master Mix (Bio-Rad, United States) on a CFX96 real-time PCR detection system (Bio-Rad).

Gene expression levels of KRT14, MKI67, and ESR1 were assessed, using GAPDH as the internal control. Each reaction was performed in triplicate. The relative expression levels were calculated using the 2^-ΔΔCt method, setting the matched tissue samples as calibrators. Primer sequences were as follows.

• KRT14: forward 5′-CAG​GAG​GCG​GAT​GAG​GTG-3′; reverse 5′-CAG​GTC​CTG​GAG​AGG​GAA​TC-3′
• MKI67: forward 5′-AGA​CCT​CCT​GAG​CCT​GAA​GA-3′; reverse 5′-GGT​TAT​GAG​GGC​AGT​GAC​TGC-3′
• ESR1: forward 5′-AGA​GGT​GCC​CTA​CTA​CCT​GG-3′; reverse 5′-CAG​ACG​AGA​CCA​ATC​ATC​AGG-3′
• GAPDH: forward 5′-GAA​GGT​GAA​GGT​CGG​AGT-3′; reverse 5′-GAA​GAT​GGT​GAT​GGG​ATT​TC-3′

Statistical analysis was performed using GraphPad Prism version 9.5 (GraphPad Software). Paired two-tailed Student’s t-tests were applied to assess differences between organoid and tissue groups, and p-values less than 0.05 were considered statistically significant. The full set of original Ct values, ΔCt, ΔΔCt, and 2^-ΔΔCt calculations is provided in Supplementary Table S3.

## 4 Result

### 4.1 Establishment of male gynecomastia organoids and histological validation

**FIGURE 7:**
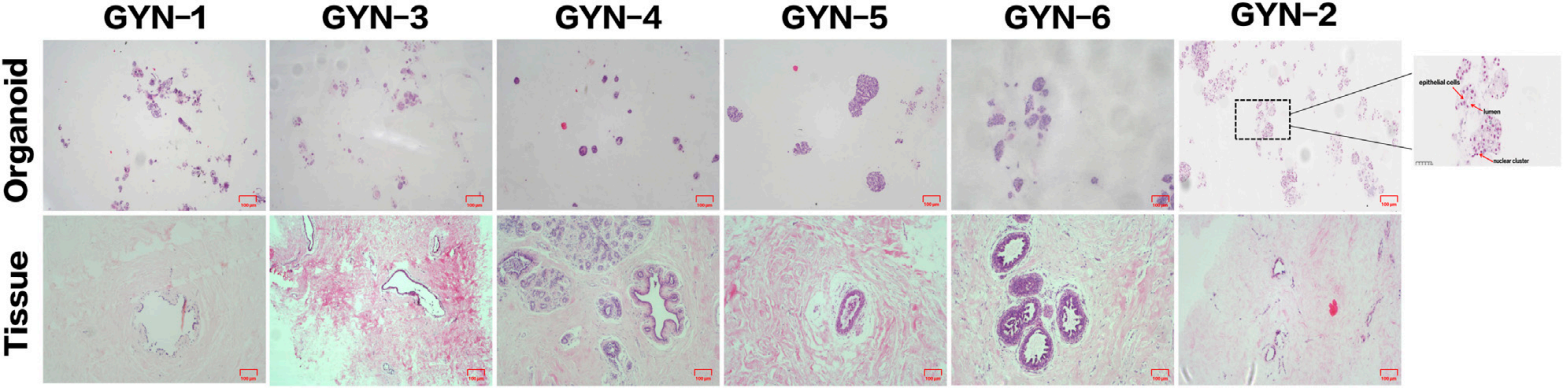
Histological comparison of organoids and matched source tissues using H&E staining. Representative H&E-stained sections from six gynecomastia cases demonstrate similar glandular architecture and epithelial organization between organoids and their corresponding tissues. A high-magnification inset (×400) from the GYN-2 organoid (boxed region, far right) highlights key structural features, including a lumen-like space, epithelial cells, and nuclear clusters, marked by arrows. Scale bars: main panels = 100 μm; inset = 50 μm.

Organoids were successfully established from six male gynecomastia patient samples (designated GYN-1 to GYN-6). The morphological fidelity of the cultured organoids to their corresponding source tissues was assessed by hematoxylin and eosin (H&E) staining (Figure 7).

The organoids displayed well-organized glandular-like structures that closely mirrored the architectural features of the original tissues. In particular, samples such as GYN-5 and GYN-6 exhibited distinct acinar arrangements, with densely packed cellular clusters and well-defined luminal spaces. The size, nuclear morphology, and chromatin patterns of organoid cells were consistent with those observed in the matched source tissues. Additional samples (e.g., GYN-2 and GYN-4) showed strong similarity in terms of cytoplasmic staining intensity, nuclear shape, and overall tissue architecture.

These findings confirm that the cultured organoids successfully replicated the key histological characteristics of male gynecomastia tissue, including ductal epithelial proliferation and preserved glandular morphology, as described in prior large-scale morphological studies of gynecomastia tissue (Prasad et al., 2022).

### 4.2 Immunohistochemical confirmation of phenotypic stability

**FIGURE 8:**
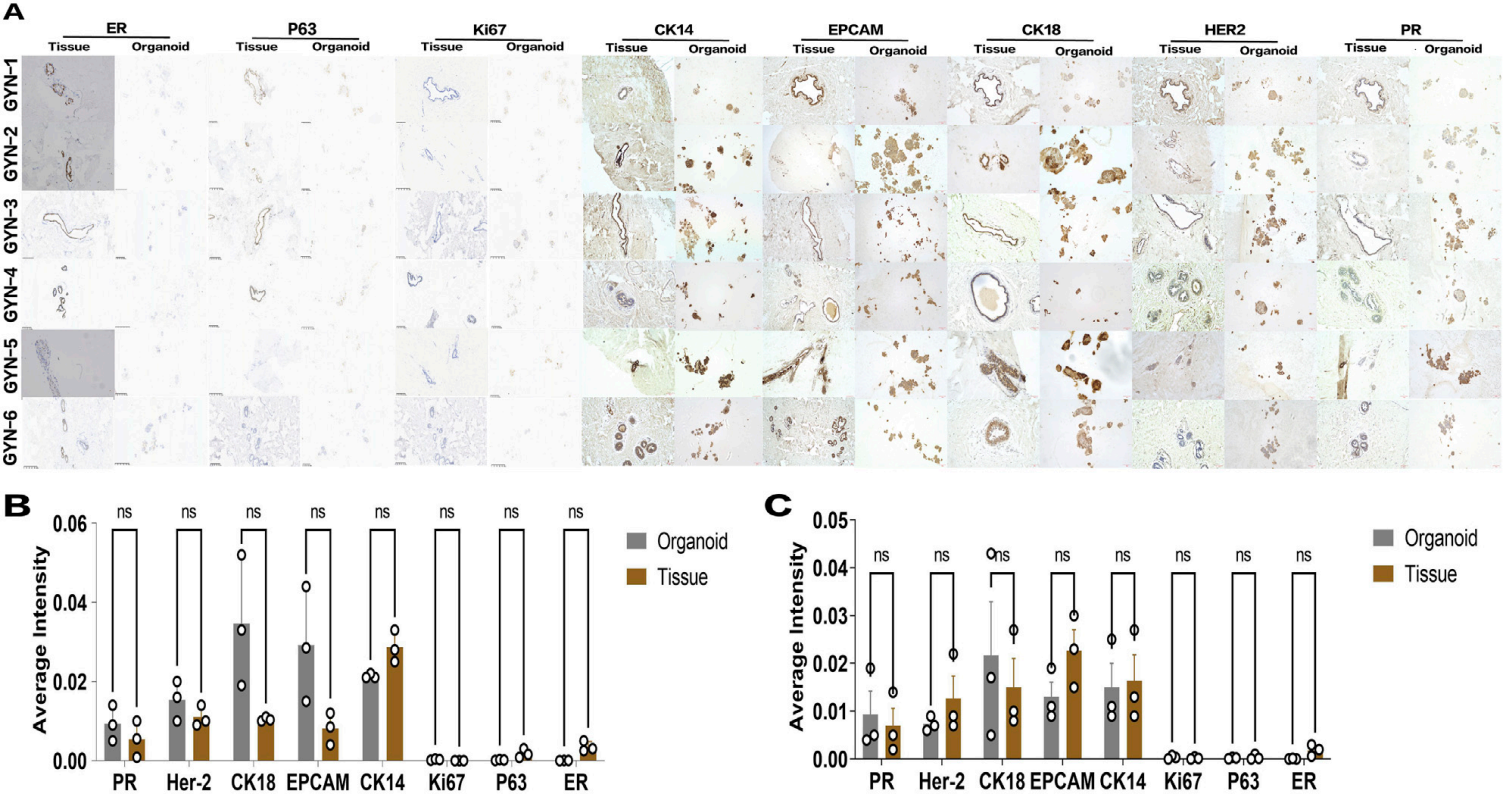
Immunohistochemical evaluation of marker expression in gynecomastia organoids and matched tissues. **(A)** Representative IHC staining for eight markers (ER, PR, Ki67, P63, CK14, CK18, HER2, EPCAM) in paraffin-embedded sections from organoids and matched tissues of six patients (GYN-1 to GYN-6). Scale bar = 100 μm. **(B,C)** Quantification of DAB signal intensity for each marker across all samples **(B)** and within the hormone-related gynecomastia subgroup **(C)**. Each dot represents the average grayscale intensity from three non-overlapping ROIs (fields of view) in a single patient sample (n = 3 per group). DAB intensities were normalized to the overall marker-specific mean across all samples to allow intergroup comparison. Values are presented as mean ± standard deviation. Comparisons were conducted using paired two-tailed t-tests in GraphPad Prism. No statistically significant differences were observed for any marker.

Immunohistochemical analysis further validated the phenotypic resemblance between organoids and their corresponding tissues (Figure 8). A panel of eight markers was evaluated, including estrogen receptor (ER), progesterone receptor (PR), HER2, cytokeratin 18 (CK18), epithelial cell adhesion molecule (EPCAM), cytokeratin 14 (CK14), Ki67, and p63.

Consistent expression patterns were observed between organoids and source tissues across all samples. Hormone receptors (ER and PR), epithelial lineage markers (CK18 and CK14), and proliferation/stemness markers (Ki67 and p63) were uniformly expressed in both compartments. Notably, there were no significant differences in expression intensity for any of the eight markers tested, suggesting robust preservation of molecular phenotypes during organoid culture.

This result underscores the stability of the organoid system and supports its use as a representative model of male gynecomastia tissue.

Notably, there were no significant differences in expression intensity for any of the eight markers tested, suggesting robust preservation of molecular phenotypes during organoid culture. Across subtypes, marker expression was generally conserved, though ERα tended to be lower in hormone-related cases.

To further illustrate inter-sample consistency, Supplementary Table S4 summarizes the organoid culture success and key marker expression (CK18, CK14, Ki67, and ERα) across all six gynecomastia patients.

### 4.3 Immunofluorescence reveals high concordance in marker expression with reduced ERα levels

**FIGURE 9:**
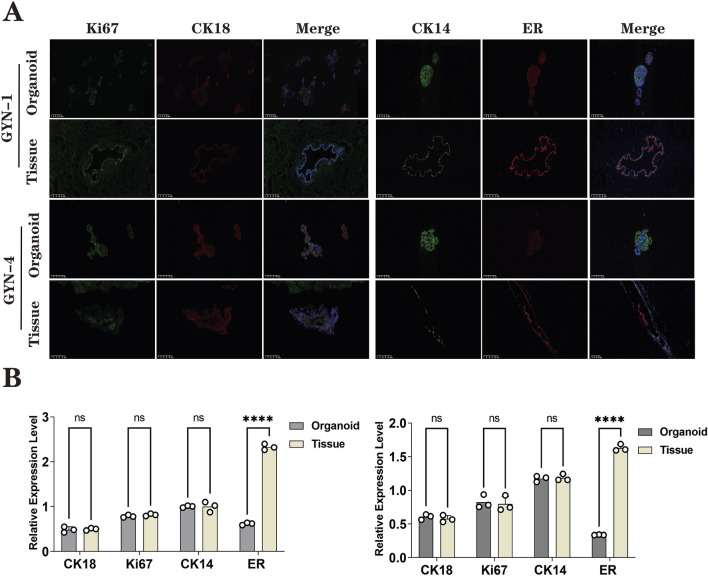
Immunofluorescence analysis of organoids and source tissues from physiological (GYN-1) and hormone-related (GYN-4) cases. **(A)** IF staining for CK18 (green), Ki67 (red), CK14 (purple), and ERα (orange), with DAPI nuclear counterstain (blue); merged images included. **(B)** Quantitative fluorescence intensity comparison between organoids and tissues. Each dot represents the fluorescence intensity of one non-overlapping field (n = 3 per group), as measured by ImageJ. Field-level data are provided in Supplementary Table S2. ERα expression showed greater variability across samples. Scale bar = 100 μm.

To further confirm molecular fidelity, immunofluorescence analysis was performed on organoids and corresponding tissues derived from a physiological gynecomastia case (GYN-1) and a hormone-related case (GYN-4) (Figure 9A). Two marker groups were evaluated.

• Group 1: Luminal epithelial marker CK18 and proliferation marker Ki67
• Group 2: Basal marker CK14 and estrogen receptor α (ERα)

Quantitative analysis of fluorescence signal intensity (Figure 9B) demonstrated high concordance between organoids and tissue for CK18, CK14, and Ki67, indicating that the organoids retained both luminal and basal cell identities as well as proliferative capacity.

However, ERα expression was markedly reduced in organoids compared to their corresponding tissues, with statistical significance (paired t-test, p < 0.0001). This suggests a possible downregulation of estrogen receptor expression under current culture conditions, which may reflect microenvironmental or hormonal differences *in vitro*.

Across all six gynecomastia cases analyzed by immunohistochemistry and immunofluorescence, the expression patterns of CK18, CK14, and Ki67 were consistently observed in both organoids and source tissues, with only minor variation in staining intensity. These results support the reproducibility of the organoid culture protocol across patients.

### 4.4 qPCR confirms transcriptional consistency between organoids and source tissues

**FIGURE 10:**
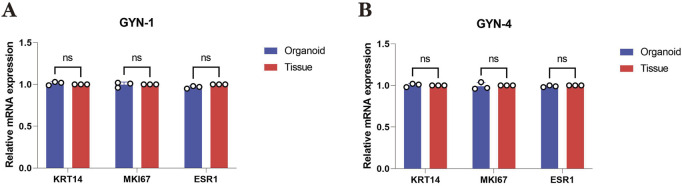
Quantitative PCR analysis of key marker genes in gynecomastia-derived organoids and matched source tissues. **(A)** Relative mRNA expression levels of KRT14, MKI67, and ESR1 in GYN-1 (physiological type) organoids and corresponding breast tissue. **(B)** Relative mRNA expression levels of the same markers in GYN-4 (hormone-related type). Gene expression was normalized to GAPDH and calculated using the 2^(-ΔΔCt) method, with matched tissue samples serving as calibrators (set to 1.00). Each bar represents the mean ± standard deviation (SD) of three biological replicates. No significant differences were observed between organoids and source tissues for any marker (paired t-test, p > 0.05). These results indicate that the transcriptional profiles of epithelial (KRT14), proliferative (MKI67), and hormone receptor (ESR1) markers were preserved in the organoid model.

To further investigate whether organoids retained the transcriptional characteristics of their source tissues, qPCR was conducted for three key markers—KRT14 (basal epithelial marker), MKI67 (proliferation marker), and ESR1 (estrogen receptor alpha)—in GYN-1 and GYN-4. As shown in Figure 10, the relative mRNA expression levels of all three genes were highly consistent between the organoids and their corresponding tissues. No statistically significant differences were observed for any marker in either case (paired t-test, p > 0.05), indicating stable transcriptional profiles across basal, proliferative, and hormone-responsive pathways. These results further support the phenotypic fidelity of the organoid model at the gene expression level.

### 4.5 Dynamic monitoring confirms growth and structural maturation

Representative brightfield images of GYN-1-derived organoids were captured over a 9-day culture period to monitor growth dynamics (Figure 5). The organoid structure became increasingly complex, with clear expansion of boundary contours and progressive development of internal microstructures. The consistent enlargement and viability of the organoid during this period demonstrate the reproducibility and structural stability of the culture system.

## 5 Discussion

This study describes the successful establishment and characterization of a patient-derived organoid model for male gynecomastia, offering a novel and practical tool to study benign male breast disease *in vitro*. Through comprehensive histological and molecular analyses, we demonstrated that these organoids recapitulate the key structural and phenotypic features of their source tissues across distinct gynecomastia subtypes.

Hematoxylin and eosin (H&E) staining revealed that organoids maintained characteristic glandular structures and nuclear morphology, especially in GYN-2, GYN-4, and GYN-5, suggesting robust preservation of microarchitectural features. These observations align with the histological features commonly described in gynecomastia, such as ductal epithelial hyperplasia and lobular-like expansion (Johnson and Murad, 2009). Immunohistochemical staining further confirmed consistent expression of basal, luminal, hormonal, and proliferative markers, including CK14, CK18, Ki-67, ER, PR, and P63, across all cases. Notably, this molecular consistency was observed in organoids from physiological, idiopathic, and hormone-related gynecomastia, supporting the reproducibility and applicability of the model across etiological backgrounds. These features are consistent with known histopathological characteristics of gynecomastia, including ductal epithelial hyperplasia, stromal fibrosis, and hormone receptor positivity, as described in previous studies (Narula and Carlson, 2014).

HER2 was included in our marker panel to help exclude early neoplastic changes and ensure that the organoid cultures represented benign tissue. Although HER2 is primarily studied in female breast cancer, occasional HER2 expression has been reported in male breast specimens, particularly in the context of hormonal imbalance or atypical hyperplasia (Giordano, 2005). Including HER2 also allowed us to confirm the absence of malignant transformation in our samples.

These findings align with previous work on female breast organoids, which demonstrated that 3D cultures retain source tissue lineage markers and architecture (Sachs et al., 2018; Dekkers et al., 2019). Our study expands upon these observations, demonstrating that gynecomastia organoids similarly preserve key phenotypic and structural characteristics, validating their potential as *ex vivo* models for male benign breast conditions. Although flow cytometry was suggested for further validation, we prioritized histology-based approaches (IHC and IF) to preserve spatial marker context and due to the limited number of organoid samples. These methods allowed robust phenotypic characterization without compromising structural integrity.

Interestingly, while immunofluorescence revealed a significant reduction in ERα protein levels in organoids, qPCR showed no significant change in ESR1 transcription. This discrepancy likely reflects post-transcriptional regulation of ERα under *in vitro* conditions. Previous studies have demonstrated that ERα protein levels decline in the absence of estrogenic stimuli, despite transcriptional maintenance of ESR1 (Söderqvist et al., 2010). This suggests that the hormonal environment and receptor degradation pathways within the culture system influence ERα protein stability.

This divergence between mRNA and protein levels may be attributed to several factors.

(1) the absence of estrogen or other hormonal cues in the current culture system,
(2) loss of stromal or paracrine signaling required for ERα stabilization, and
(3) selection bias introduced by tissue dissociation or long-term culture.

Despite this limitation, the preservation of ESR1 transcriptional activity highlights the model’s retained potential for hormonal modulation, pending optimization of the *in vitro* microenvironment.

Importantly, this is the first organoid system established from male gynecomastia patients, filling a critical gap in disease-specific *in vitro* modeling. While organoids have been extensively developed for female breast tissues and malignancies, benign male breast conditions have been largely overlooked. Our results establish gynecomastia-derived organoids as a stable and scalable platform for mechanistic studies, hormone response analyses, and future therapeutic testing.

## 6 Limitations

Several limitations should be acknowledged in this study.

1. Lack of normal male breast controls: Due to ethical and clinical constraints, healthy male breast tissue was not available for comparison. This limits the ability to distinguish disease-specific features from normal male mammary epithelium.
2. Limited sample size: Although only six cases were included, they represent the major clinical subtypes of gynecomastia—physiological, idiopathic, and hormone-related—which enhances the representativeness of the model.
3. Absence of functional hormone assays: No *in vitro* estrogen or androgen stimulation experiments were performed to evaluate endocrine responsiveness, although such studies are ongoing.
4. Limited molecular profiling: While qPCR validation of key markers was conducted, transcriptomic analyses such as RNA-seq were not performed, primarily due to limited tissue material.
5. Lack of protein quantification via immunoblotting: Western blotting was not feasible given the limited yield of organoid cultures; however, this will be addressed in future studies through bulk or single-cell proteomics.
6. Incomplete lineage characterization: Markers of progenitor and developmental states (e.g., SOX9, ALDH1A1) were not assessed and should be incorporated into future profiling of the model.

## 7 Future directions and applications

To improve the physiological relevance of the model, future efforts should focus on modifying the culture system through hormone supplementation (e.g., estradiol or androgens), co-culture with stromal or immune components, and dynamic hormone cycling. These adjustments may help restore ERα protein expression and better simulate the native breast microenvironment.

In addition, integrating bulk or single-cell transcriptomics will enable deeper exploration of cell-type diversity, lineage commitment, and inter-subtype variability in gynecomastia. Functional hormone assays are also in progress and will clarify the model’s endocrine responsiveness.

From a translational perspective, patient-derived gynecomastia organoids offer a promising platform for personalized medicine. They can be used to test individual hormone sensitivity, screen endocrine modulators, and evaluate potential environmental disruptors. This model could be integrated into preclinical pipelines for benign male breast disorders, enabling mechanism-guided therapeutic strategies.

## 8 Conclusion

In summary, we successfully established a stable and phenotypically faithful organoid culture system from male gynecomastia tissues. These organoids recapitulate the structural architecture and key molecular features of their source tissues, including basal, luminal, proliferative, and hormonal markers.

Although reduced ERα protein expression was observed under standard culture conditions, ESR1 gene expression remained consistent, suggesting that the system retains transcriptional responsiveness to hormonal pathways. This indicates potential for future functional enhancement through culture optimization.

Our work provides the first organoid model specific to male gynecomastia and expands the organoid field to encompass benign male breast conditions. This model offers a valuable experimental platform for studying gynecomastia pathogenesis, evaluating hormonal modulation, and advancing personalized therapeutic approaches.

## Data Availability

The original contributions presented in the study are included in the article/Supplementary Material, further inquiries can be directed to the corresponding author.
