# Supplementary material for: A protocol for organoids from the gynecomastia patients
Source: Front Bioeng Biotechnol. 2025 Aug 13;13:1593368. doi: 10.3389/fbioe.2025.1593368 (PMC12381642; doi:10.3389/fbioe.2025.1593368)
Supplement: Supplementary file 3 [file Table1.docx]

| **REAGENT or RESOURCE** | **SOURCE** | **IDENTIFIER** |
| --- | --- | --- |
| Antibodies |  |  |
| CK14 | ZSGB-BIO | ZM-0540 |
| CK18 | Abcam | ZM-0315 |
| Her2 | Origene | TA503759S |
| EpCAM | ZSGB-BIO | ZM-0315 |
| P63 | ZSGB-BIO | ZM-0315 |
| Ki-67 | ZSGB-BIO | ZM-0315 |
| PR | Cell signaling | 3157 |
| ER | Origene | TA503759S |
| Chemicals, peptides, and recombinantproteins | | |
| Mammary Organoid Culture Medium |  | OM18N |
| Tissue Digestgion Solution |  | OM41 |
| Mild Cell Digestion Solution |  | OM42 |
| Digestion Neutralization |  | OM43 |
| Matirgel |  | OM21 |
| Tissue Preservation | | OM45 |
| Organoid Freezing Medium |  | OM46 |

| **MATERIAL AND EQUIPMENT** | **Parameter** |
| --- | --- |
| Centrifuge | 15ml、50ml |
| Cell Strainer | 100μm |
| Low Adhesion Cell Culture Plates | 24-well、96-well |
| Pipette Tips | 10μl、200μl、1000ul |
| Biosafety Cabinet | CalssII |
| Incubator with Humidity and Gas Control |  |
| Low-Speed Centrifuge | With horizontal rotor |
| Pipettes | 1ml、200μl、10μl |
| Cell Counter or Automated Cell Counter | With horizontal rotor |
| Inverted Microscope |  |
| Freezer | -20°C |
| Refrigerator | (2 - 8°C) |
| Eppendorf Tubes | 1.5ml |
| Ophthalmic Scissors |  |
| Ophthalmic Forceps |  |
| Culture Dish | 10cm |
| Dulbecco's Phosphate-Buffered Saline (DPBS) |  |
| Sterile Surgical Blade Handle and Blades |  |

**Table S1:** Experimental Reagents and Consumables
